# High quality draft sequences for prokaryotic genomes using a mix of new sequencing technologies

**DOI:** 10.1186/1471-2164-9-603

**Published:** 2008-12-16

**Authors:** Jean-Marc Aury, Corinne Cruaud, Valérie Barbe, Odile Rogier, Sophie Mangenot, Gaelle Samson, Julie Poulain, Véronique Anthouard, Claude Scarpelli, François Artiguenave, Patrick Wincker

**Affiliations:** 1 CEA, DSV, Institut de Génomique, Genoscope, 2 rue Gaston Crémieux, CP5706, 91057 Evry, France; 2 CNRS, UMR 8030, 2 rue Gaston Crémieux, CP5706, 91057 Evry, France; 3 Université d'Evry, 91057 Evry, France

## Abstract

**Background:**

Massively parallel DNA sequencing instruments are enabling the decoding of whole genomes at significantly lower cost and higher throughput than classical Sanger technology. Each of these technologies have been estimated to yield assemblies with more problematic features than the standard method. These problems are of a different nature depending on the techniques used. So, an appropriate mix of technologies may help resolve most difficulties, and eventually provide assemblies of high quality without requiring any Sanger-based input.

**Results:**

We compared assemblies obtained using Sanger data with those from different inputs from New Sequencing Technologies. The assemblies were systematically compared with a reference finished sequence. We found that the 454 GSFLX can efficiently produce high continuity when used at high coverage. The potential to enhance continuity by scaffolding was tested using 454 sequences from circularized genomic fragments. Finally, we explore the use of Solexa-Illumina short reads to polish the genome draft by implementing a technique to correct 454 consensus errors.

**Conclusion:**

High quality drafts can be produced for small genomes without any Sanger data input. We found that 454 GSFLX and Solexa/Illumina show great complementarity in producing large contigs and supercontigs with a low error rate.

## Background

Whole-genome sequencing has profoundly impacted the field of prokaryotic genetics since its first demonstration[1]. Almost all of the economically and medically important microbes have had at least one representative with their genome sequenced. This achievement was first seen as the main goal of bacterial genomics, but is now strongly challenged by two observations. First, most microbial diversity is represented by uncultivated organisms, so the genomes sequenced today only represent a small fraction of the microbial gene space. Second, the variability between members of the same bacterial "species" can be very high in terms of gene content[2,3]. Therefore, the definition of the proteome for a defined taxon may necessitate the sequencing of numerous related genomes. New technologies are therefore needed to sequence a larger amount of prokaryotic genomes than previously thought. A number of new methods have reached the commercialization stage in the last few years. They are based on principles that are different from dideoxy termination and electrophoretic separations, as in the Sanger method[4,5]. As such, they display different error rates and types, and produce assemblies with different characteristics. The most commonly used method[6], that make use of highly parallelized pyrosequencing, has an inherently higher error rate around tracts of mononucleotides[7,8]. This translates into higher insertion-deletion errors in assembly consensus, and in-frame stop codons in genes.

For *de novo *sequencing, these technologies have two main drawbacks beyond the sequencing error issue. First, they have been developed in the framework of the resequencing of the human genome, and thus produce mostly short reads that are useful for detecting substitution polymorphisms against a reference genome, but are more difficult to use for *de novo *assembly of a new genome. Second, their initial implementation permitted only un-paired sequences. The presence of links between two reads is a major element for *de novo *sequencing[9], enabling both the linkage of different contigs separated by a sequence gap, and the construction of robust contigs by detection of assembly problems due to repeated elements[10]. For these reasons, the accuracy and continuity of assemblies obtained with new sequencing technology data were lower than those traditionally obtained with the Sanger approach. Recent improvements of the new technologies brought the promise of a better final product for WGS projects. Here, we evaluated how assemblies made with such improvements compare with assemblies produced with Sanger data, and how a mix of Roche/454 and Solexa/Illumina technologies performed in whole-genome sequencing of a reference bacterial genome.

## Results

For testing the efficiency of different approaches in bacterial genome assembly, we chose the gamma-proteobacterium *Acinetobacter baylyi *as a test-case. A finished version of the genome has already been produced[11], and posterior projects of global gene disruptions have led to resequencing almost all genes, thus providing a very error-free reference[12]. Additionally, this genome was sequenced in our lab, thus permitting reanalysis of any incongruencies between the reference assembly and the versions generated with the new data sets. *Acinetobacter baylyi *has a genome size of 3.6 Mb with a GC content of about 40%.

### Sanger assembly

We first generated a draft assembly with Sanger data at 7.4× coverage (see Methods). The characteristics of this assembly are described in Table 1. The error rate of this draft sequence was calculated by comparison with finished version[11] at about 1 per kb. The draft version lacked 181 kb of the final version, reflecting lack of assembly for some repeated sequences, and potential cloning bias. The use of two libraries with different-sized insert allowed construction of two supercontigs covering the circular genome. As this type of assembly is typical for draft genomes obtained with Sanger sequencing, we will use it as a reference for comparison with drafts produced with the new techniques.

**Table 1:** Characteristics of the assemblies with different data inputs

	Coverage	Contigs (number)	Contigs (N50)	Scaffolds (number)	Scaffolds (N50)	Assembly size (% of reference)	Mis-assemblies	Total errors	Substitutions	Insertions/Deletions
Sanger	7.4x	173	39 kb	2	2.2 Mb	3.417 Mb (95%)	0	3442	2494	948

Unpaired 454	20x	119	48.7 kb	119	48.7 kb	3.542 Mb (98%)	0	420	67	353

Unpaired + paired 454	25x	119	58.2 kb	10	1 Mb	3.544 Mb (98%)	0	431	75	356

unpaired + paired 454 with Illumina/Solexa GA1	25× and 50×	119	58.2 kb	10	1 Mb	3.544 Mb (98%)	0	163	71	92

### 454-only metrics

**Figure 1:**
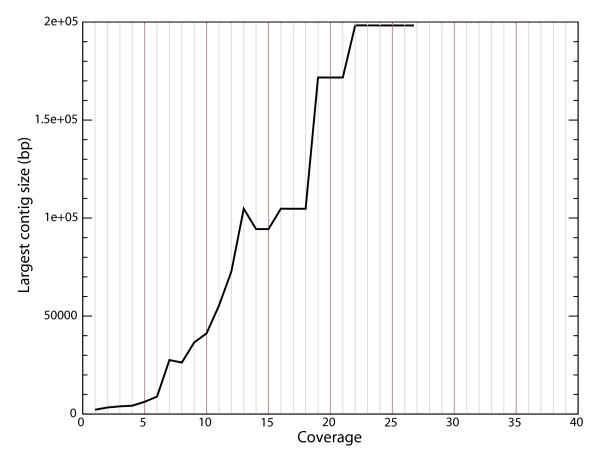
**Largest contig size for each Newbler assembly from a coverage of 1× to 27×**.

**Figure 2:**
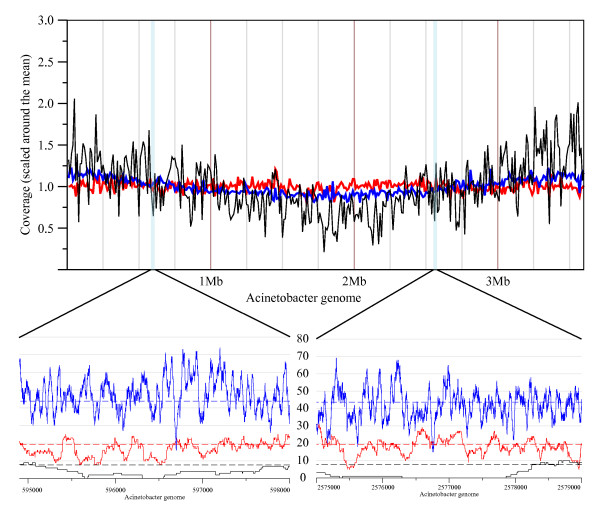
**Genome coverage (scaled around the average coverage) at a resolution of 10 Kb (upper graph) along the entire genome of Acinetobacter, and genome coverage of two genomic regions at the base level (two bottom graphs)**. The black curve is the Sanger reads coverage (the average coverage is the black dashed line), blue lines are Solexa reads coverage and red lines GSFLX reads coverage.

We performed assemblies of *A. baylyi *with increasing coverage using Roche/454 GSFLX data. We observed a plateau for the assembly metrics at about 20–25 genome equivalents (see Figure 1, and additional files 1 and 2). The parameters of the 20× assembly are shown in Table 1. Due to its higher coverage, the error rate is lower than for the draft Sanger assembly (1 error per 8.4 kb). The number of bases covered is also superior. This confirms previous reports that 454/Roche technology is able to produce high quality draft assemblies if used at sufficient redundancy[13]. It is also apparent that the errors are biased towards insertion-deletions (Table 1). We checked these errors visually and confirmed that they reflect essentially misincorporation in or around homopolymeric sequences. Finally, the low error rate is also due to relatively uniform coverage (see Figure 2), reflecting the absence of bias introduced by cloning as in the Sanger method.

### Paired-end sequencing

We next explored the possibility of obtaining more continuity with GSFLX data by sequencing a "paired" library using the Roche/454 protocol (see Methods). We produced about 5× supplemental coverage with a "paired" library using circularized fragments of 3 kb[14], of which about one-fourth were detected as paired sequences by the Newbler assembler (see Additional File 3). The other three-fourths are used in the assembly, but contained no pairing information since they are not constituted of two sufficiently-sized regions separated by the spacer. The number of contigs did not change from the 20× unpaired distribution, reflecting that the main impact of the 454 paired reads is the bridging of contigs. We obtained 10 scaffolds. When compared with the reference sequence, all gap positions appeared in repetitive regions that are more than 3 kb long. This indicates that at this coverage, all possible supercontiging has been automatically achieved. An obvious way of improving the assembly will be the construction of paired libraries from circularized fragments of larger size than 3 kb. Our results indicate that with appropriate sizing of such libraries, most bacterial genomes may be obtained as a collection of very few supercontigs using 454/Roche data only.

### Use of Solexa data to correct the consensus sequence

**Figure 3:**
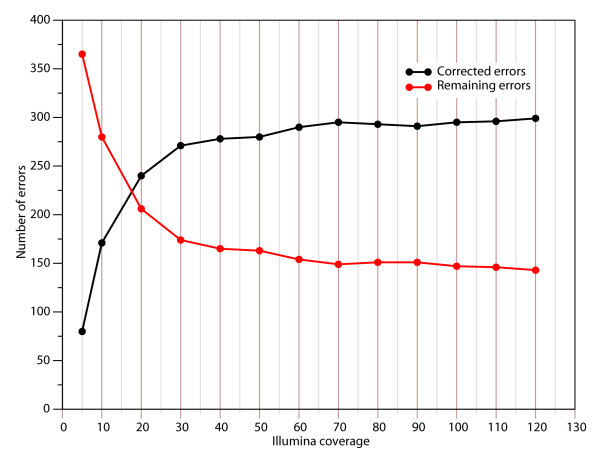
**Number of corrected and remaining errors after correction with different coverage of Solexa reads**.

One way to improve the GSFLX assembly is to complement it with another type of data with a different bias in error type. We generated short-read sequences on a Solexa/Illumina GA I genome analyser and determined the error distribution. As previously reported, errors consist mainly (98.8% in our data set) of mismatches (e.g. bases called as another base) [15-17]. This data type seems well adapted for correction of GSFLX errors, which are mainly indels. The Solexa reads were mapped at increasing coverage onto the 25× GSFLX assembly using SOAP software[18], which allows alignments with gaps. We found that a coverage of about 50× enabled correction of 268 of the initial 431 errors (62%; Figure 3 and Table 2). Additional coverage may be used to correct a few more errors, but the method reached a plateau rapidly (for example we only correct 16 additional errors by raising coverage from 50 to 100x; Table 2). We examined all the remaining 163 errors manually to determine why our procedure was unable to correct them. Fifty-one of these errors were attributed to errors in the original consensus sequence or to the presence of variations occurring during cultivation in one of the experiments (the DNA source for the Sanger-based sequencing came from a different cultivation than that used for the high-throughput experiments). The 112 other errors are found in repetitive regions or low coverage (with Solexa reads) regions (contigs extremity).

**Table 2:** Remaining errors after correction using Solexa/Illumina reads with different coverage

Solexa Coverage	Remaining Substitutions	Remaining Insertions	Remaining Deletions
5x	77	127	161
10x	75	95	110
20x	73	59	74
30x	73	48	53
40x	71	46	48
50x	71	46	46
60x	71	43	40
70x	70	43	36
80x	70	43	38
90x	70	43	38
100x	69	40	38
110x	69	39	38
120x	69	38	36

**Figure 4:**
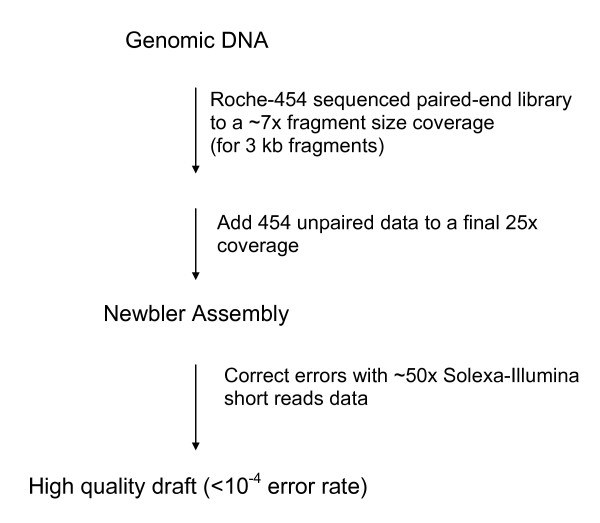
**Proposed optimized strategy for sequencing a prokaryote genome with Roche-454 and Solexa/Illumina data**.

Taken together, these improvements described previously led to the generation of a draft sequence for *A. baylyi *that covered all non-repetitive regions with an error rate of < 3.10^-5^. Most of the sequence is of even better quality, since the remaining errors clearly concentrate in contig ends (see Additional file 4). We can propose a simple scheme to produce a draft sequence for any prokaryotic genome efficiently and cost-effectively (Figure 4) : The first step is the generation of paired-end reads using GSFLX technology to obtain about 10× bridge coverage (this may correspond to different sequence coverage depending on the quality of the paired-end library). Then, additional sequences are produced using unpaired GSFLX data to reach 20–25× final coverage. The raw data is assembled using Newbler, and 50× coverage Solexa reads are used to correct the GSFLX errors using the procedure described before.

**Figure 5:**
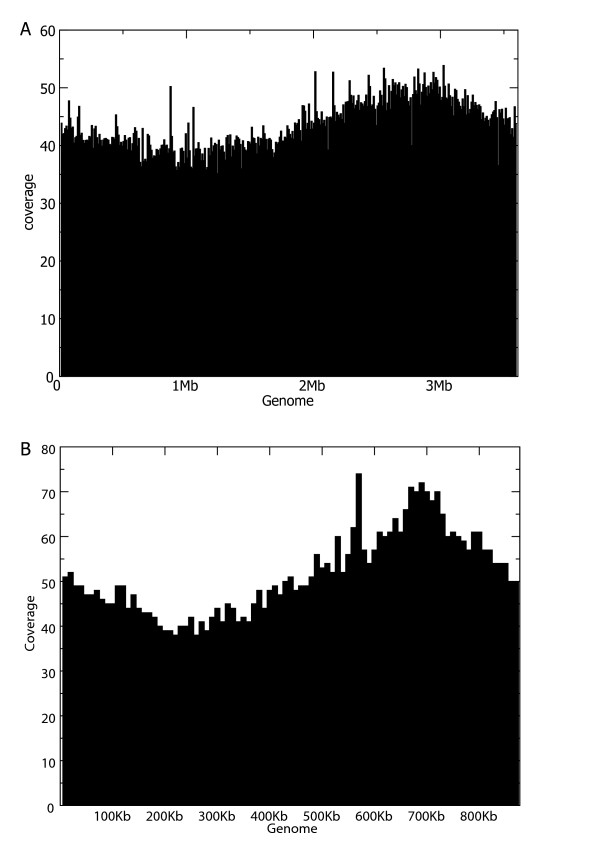
**(A) Coverage of the Acinetobacter genome with Solexa/Illumina reads.** The replication origin was replaced around 3 Mb. (B) Coverage of the Mycoplasma agalactiae genome with Solexa/Illumina reads. The replication origin was replaced around 700 Kb.

One of the expected outcomes of a prokaryote genome sequencing project is the localisation of the origin of replication of the replicon(s). Different non-experimental approaches exist, the most widely used being the localization of GC skew in the sequence[19]. We found that high throughput sequencing data can be used for this purpose, as recently discussed for ligation sequencing[20]. In particular, the Solexa data generated from an exponentially growing culture presents a distribution bias, with more sequences near the replication origin and fewer near the terminus (Fig 5B). This method may be generalized to other organisms, since we easily reproduce the same pattern with another genome[21] (Fig 5A). Although this only localizes the region containing the replication origin roughly, it may serve as a first approach to delimit a reduced interval. Other methods, like GC-skew analysis may then be applied on this limited region to localize the origin more precisely.

## Discussion

The recent availability of new sequencing technologies has fueled enormous expectation for the rapid and cost-effective determination of the sequence of small genomes. The usefulness of such genome draft sequences will be dependent on their quality, principally in terms of their error rates and contiguity. In this study, we tested the effect of mixing two different data types (454 and Solexa/Illumina) to deliver a high quality draft for a bacterial genome. There is no easy way to co-assemble efficiently these two data sets, so we devised a method that assembles the genome first using the longest reads, then corrects the remaining errors by aligning the shortest sequences to the consensus. We were able to obtain a high accuracy consensus, together with a low number of total scaffolds using this approach. The improvement over the use of a single technology is evident, and may be attributed to the different error types in each technology. We determined the approximate level of redundancy required to obtain optimal results as 25× (for 454 data assembled with Newbler) and 50× (for Solexa data). These numbers are indicative, as they may be slightly different according to the nature of the target genome. Although these numbers will evolve as these technologies develop longer read lengths and lower error rates, as well as because of assembly software improvements, this constitutes a significant decrease in the cost of a prokaryotic genome sequence compared with Sanger technology. We evaluate the total cost of this experiment as about one-third of the total cost of Sanger sequencing. Future improvements are expected to make this difference even greater.

As the number of genome sequences increases, it will be more and more difficult to finish such a large amount of draft sequences. It is therefore essential that the standard quality of a draft sequence remain high, and the method described in this paper may contribute to this objective. The main difficulty encountered in correcting all errors in the initial assembly was the lack of Solexa reads mapping uniquely at certain locations. This may be improved as the read length of Solexa data increases, as expected in the coming months. At the end of the procedure described here, we found that most remaining gaps are due to repetitive sequences. This can be improved in future versions of the assemblers that can fill gaps using paired-end data anchored on one side on single-copy sequences. These methods have been successfully employed with Sanger data [22-25], and offer the potential advantage of obtaining almost finished sequences without directed finishing, as soon as they are adapted to the new type of paired data produced with the new technologies.

## Conclusion

The combination of two technologies (454 GSFLX and Solexa/Illumina) allows production of high-quality drafts of at least a comparable quality to those obtained with Sanger data. The method presented in this study is based on available software and protocols, and may be readily implemented in many labs. Using this procedure, and with ongoing developments in assembly methods, we can expect that the advent of the New Sequencing Technologies may provide a wealth of genome sequences without compromising their overall accuracy and contiguity. This will augment their usefulness for comparative analyses.

## Methods

### Sanger sequencing

Two libraries were constructed from *A. baylyi *genomic DNA: one with 3.6 kb-inserts in the high-copy plasmid vector pcDNA2.1 after DNA shearing, and one 19.6 kb-inserts in the BAC vector pBeloBAC11 after DNA digestion with Sau3A. We obtained 24,750 and 15,739 quality-trimmed sequences for the plasmid and BAC libraries respectively. The reads were assembled using Arachne[25].

### 454 GS FLX sequencing

A library of single stranded DNA fragments was obtained from *A. baylyi *nebulized DNA according to Roche/454 standard procedures. A total of 390,596 individual reads were assembled using Newbler (20× coverage).

For the paired library, we purified 3 kb fragments after shearing using a Hydroshear device. These fragments were joined to a biotinylated linker and circularized according to Roche/454 standard procedures. After nebulization and purification of the linker-containing fragments, we amplified the library using the bead/emulsion protocol, and sequenced the products on the GS FLX sequencer. A total of 71,387 sequences were obtained after quality trimming. Of those, 18,774 were recognized as paired by the Newbler assembler. The 71,387 were assembled with 296,433 unpaired reads from the previous experiment to reach 25× coverage.

### Solexa/Illumina sequencing

The genomic DNA was unidirectionally sequenced on a Solexa/Illumina Genome Analyser I using standard procedures. The sequences were 36 bases long. A total of 12,248,948 reads passed the filter, giving a total coverage of about 123 genome equivalent.

### Assessment of error rates

The assemblies produced were compared to the reference sequence of *Acinetobacter*[11], and differences between the two versions were detected. Each contig of a given assembly was mapped onto the reference sequence using nucmer[26] with default parameters. We only retained the best match for each contig, and the resulting alignment was parsed to check for mismatches, insertions and deletions.

### Automatic error corrections with Solexa/Illumina reads

Short read sequences were aligned on the assembly using the SOAP software[18] using a seed size of 12 bps and a maximum gap size allowed on a read of 3 bps. Only uniquely mapped reads were retained. Each difference was then considered and kept only if it met the following three criteria: (1) error is not located in the first 5 bps or the last 5 bps, (2) the quality of the considered bases, the previous and the next one are above 20, and (3) the remaining sequences (before and after) around the error are not homopolymers (to avoid misalignment at boundaries). Next stage pile up errors located at the same position, particularly errors that occurred inside homopolymers (since two reads that tag the same error can report different positions). Finally, each detected error was corrected if at least three reads detected the given error and 70% of the reads located at that position agree.

Since we only allow reads uniquely mapped and reads mapped with a maximum of two mismatches and three indels, several regions were devoid of Solexa tags. In a first step, one or several errors might be corrected, and if we iterate the strategy again, regions that were devoid of Solexa reads could now be covered. We therefore decided to iterate the previous strategy until no new errors were found. For example, at 50× coverage 4 cycles were required (the 1^st ^cycle has corrected 263 errors, the second 14, the third 2 errors and the fourth and last one, no errors).

Although only three concordant reads are needed to correct a given error, the optimal result is obtained using 50× of Solexa reads. Firstly, around 10% of the initial reads cannot be mapped (due to error rate, repetitive regions, quality of reads, etc.). Next, the 3 previous criteria filter out a high proportion of mapped bases. For example, starting with 50× of Solexa reads (see Table 3 for details), criterium (1) eliminates 10 bases per read (and therefore around 30% of the coverage) and criterium (2) eliminates roughly 1/3 of the 26 remaining bases. So the usable coverage falls to 20x. Moreover, criterium (3) discards a high proportion of reads that cover homopolymers and only one of their single-copy contiguous sides, since the alignments are less reliable there. The errors in homopolymers are corrected using mostly reads that can be anchored on both sides.

**Table 3:** Evolution of the read coverage during the process of errors correction (using initially 50× of Solexa reads leads to a usable coverage of around 17x)

Sequenced reads			Uniquely mapped reads			Filtered reads		
Number of reads	Number of bases	Genome coverage	Number of reads	Number of bases	Genome coverage	Number of reads	Number of bases	Genome coverage

5.000.000	180.000.000	50,0x	4.543.370	163.561.320	45,5x	3.497.539	60.680.570	16,9x

## Authors' contributions

JMA and PW conceived the study. JMA performed bioinformatic analyses with GS and OR. JMA, VB, SM, OR, GS, VA, FA, CS and PW analysed data. CC and JP provided reagents. JMA and PW wrote the paper.

## Supplementary Material

Additional file 1 **Supplementary Figure**1. Number of large contigs in Newbler assemblies at different coverages. Click here for file

Additional file 2 **Supplementary Figure**2. N50 size in Newbler assemblies at different coverages. Click here for file

Additional file 3 **Supplementary Figure**3. Distribution of fragment size from paired-end 454 data. Click here for file

Additional file 4 **Supplementary Figure**4. Error distribution inside contigs before and after using Solexa reads for corrections. Click here for file
