# Supplementary figures and images for: High quality draft sequences for prokaryotic genomes using a mix of new sequencing technologies
Source: BMC Genomics. 2008 Dec 16;9:603. doi: 10.1186/1471-2164-9-603 (PMC2625371; doi:10.1186/1471-2164-9-603)

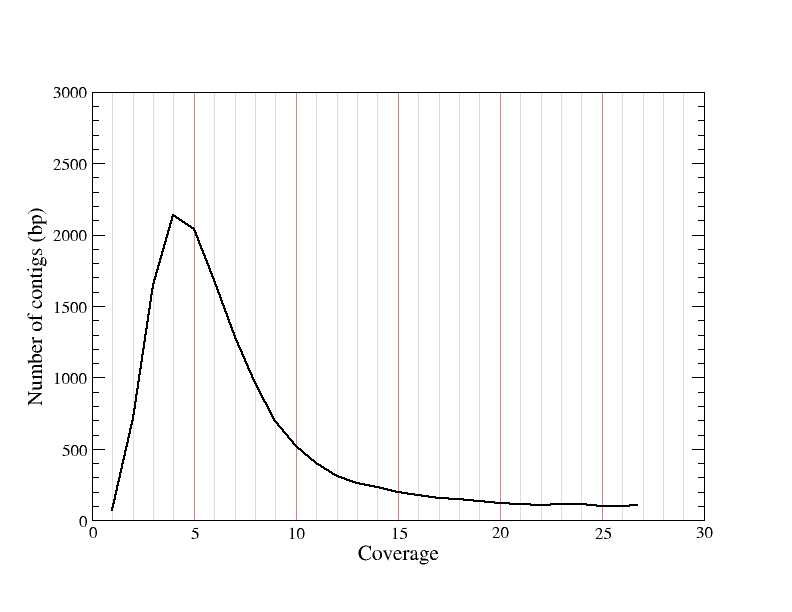


Sup Figure 1 :*Number of large contigs in Newbler assemblies at different coverages.*

Supplement: Additional file 1 — Supplementary Figure1. Number of large contigs in Newbler assemblies at different coverages. [file 1471-2164-9-603-S1.doc]

**
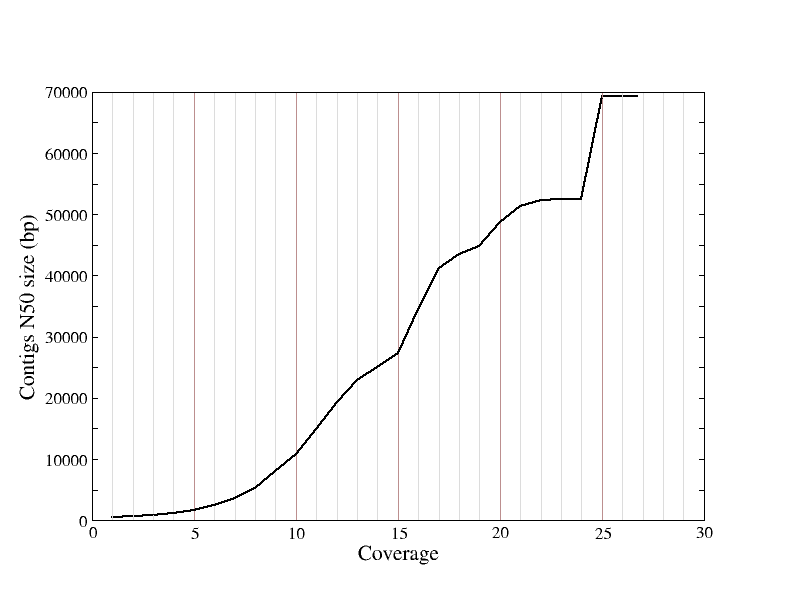
**

Sup Figure 2 :*N50 size in Newbler assemblies at different coverages.*

Supplement: Additional file 2 — Supplementary Figure2. N50 size in Newbler assemblies at different coverages. [file 1471-2164-9-603-S2.doc]

**
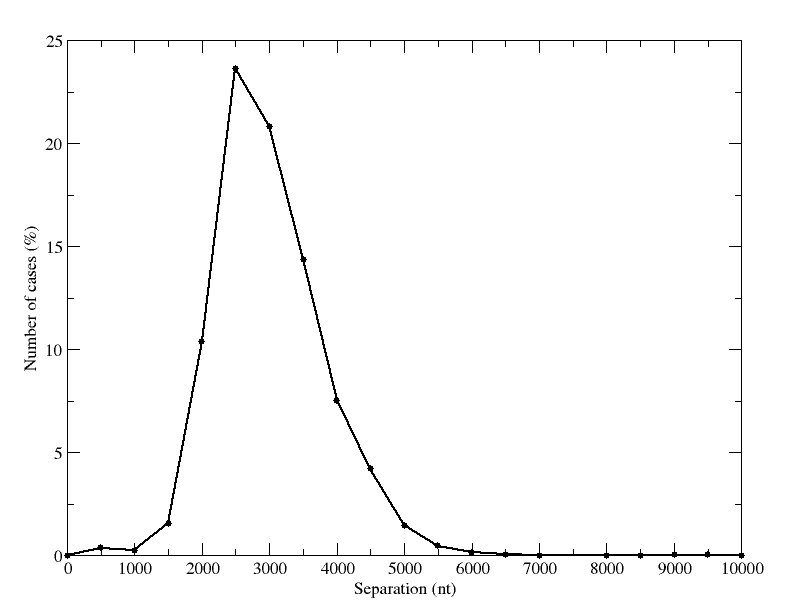
**

Sup Figure 3 :*Distribution of fragment size from paired-end 454 data.*

Supplement: Additional file 3 — Supplementary Figure3. Distribution of fragment size from paired-end 454 data. [file 1471-2164-9-603-S3.doc]
