# Supplementary material for: High quality draft sequences for prokaryotic genomes using a mix of new sequencing technologies
Source: BMC Genomics. 2008 Dec 16;9:603. doi: 10.1186/1471-2164-9-603 (PMC2625371; doi:10.1186/1471-2164-9-603)
Supplement: Additional file 4 — Supplementary Figure4. Error distribution inside contigs before and after using Solexa reads for corrections. [file 1471-2164-9-603-S4.doc]

A


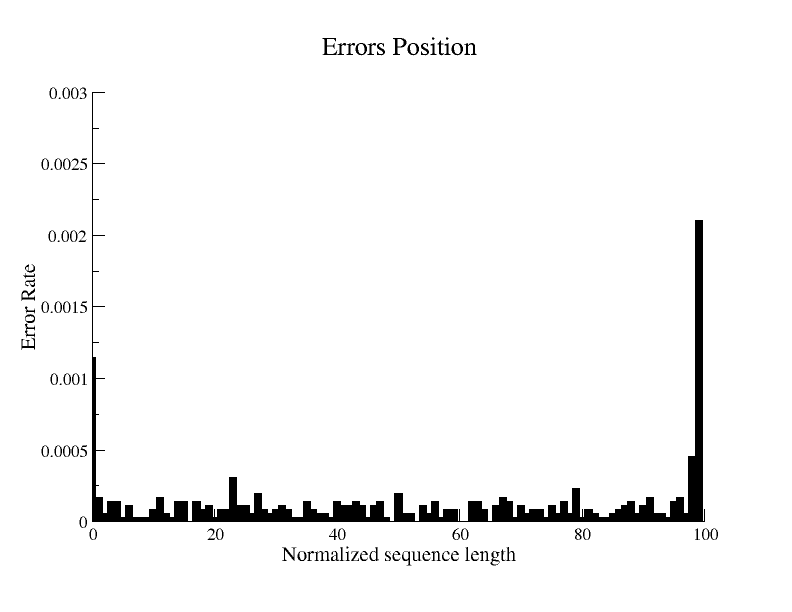


B


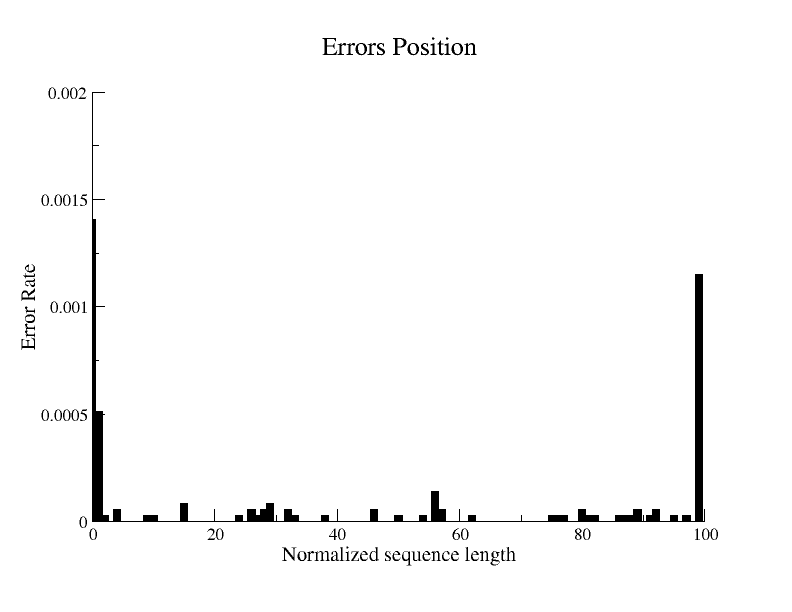


Sup Figure 4 : *Error distribution inside contigs before (A) and after (B) using Solexa reads for corrections.*
